# Effects of Toxic Concentrations of Cadmium, Lead, or Zinc on Leaf Morphology, Anatomy and Calcium Oxalate Content in Metallicolous and Non-Metallicolous Ecotypes of *Dianthus carthusianorum* L.

**DOI:** 10.3390/plants15010157

**Published:** 2026-01-04

**Authors:** Izabela Borkowska-Drela, Marcin Domaciuk, Ewa Szczuka, Jaco Vangronsveld, Małgorzata Wójcik

**Affiliations:** 1 Department of Plant Physiology and Biophysics, Institute of Biological Sciences, Maria Curie-Skłodowska University, 19 Akademicka, 20-033 Lublin, Poland; izabelaborkowska4@gmail.com (I.B.-D.); jaco.vangronsveld@mail.umcs.pl (J.V.); 2 Centre for Environmental Sciences, Hasselt University, Agoralaan Building D, 3590 Diepenbeek, Belgium; 3 Department of Cell Biology, Institute of Biological Sciences, Maria Curie-Skłodowska University, 19 Akademicka, 20-033 Lublin, Poland; marcin.domaciuk@mail.umcs.pl (M.D.); ewa.szczuka@mail.umcs.pl (E.S.)

**Keywords:** *Dianthus carthusianorum*, adaptation, anatomical structure, metal tolerance, calcium oxalate crystals, cystoliths

## Abstract

Tolerance to metals develops independently across plant species and even among populations of the same species under strong environmental pressure. This study compares the morphology and leaf anatomy of *Dianthus carthusianorum* L. originating from a Zn–Pb waste dump (metallicolous ecotype, M) and from unpolluted areas (non-metallicolous ecotype, NM), exposed to toxic concentrations of Cd, Pb, or Zn under chronic (field) and acute (hydroponic) metal stress. The aim was to identify leaf anatomical adaptations that support growth of the M ecotype in metal-polluted environments and to assess structural changes induced by acute exposure in both ecotypes. In both ecotypes, metal exposure caused alterations of mesophyll cells and the formation of abundant calcium oxalate (CaOx) crystals. Two oxalate forms were determined: insoluble (CaOx crystals) and soluble oxalates, with the former predominating. Following metal treatment, the M ecotype accumulated nearly twice as much of both forms as the NM ecotype, indicating a key role of oxalates in metal detoxification via precipitation of excess metal ions as metabolically inactive CaOx. Interestingly, elevated CaOx levels were also observed in M ecotype leaves grown under control (no metal application) conditions, suggesting a genetically fixed adaptation to metal-rich environments.

## 1. Introduction

Excessive concentrations of metals in soils are a widespread problem worldwide, arising from both natural processes and anthropogenic activities [1]. Among human-driven factors, mining and ore processing, industrialization, vehicular traffic, and inappropriate agronomic practices are the most significant contributors. Because metals cannot be degraded by natural processes, they persist indefinitely in the environment after their release, exerting detrimental effects on all components of ecosystems, including microorganisms, plants, animals, and humans [2].

The effects of metals on plants vary and depend on both the plant species and the type and concentration of the element. Mercury (Hg), lead (Pb), and cadmium (Cd) are of particular concern because they have no known physiological functions in plants and are toxic even at relatively low concentrations [3]. However, essential metallic elements, such as zinc (Zn), nickel (Ni), cobalt (Co), and chromium (Cr), can become harmful when present in excessive amounts [2,4]. Metals are taken up and accumulated in different plant organs, where they can exert negative effects at the morphological, anatomical, biochemical, and molecular levels. They interfere with photosynthesis and respiration, disturb the cellular balance between pro-oxidants and antioxidants, impair cell elongation and division, and compromise the integrity of cellular organelles [5,6]. These disturbances lead to altered root and leaf anatomy and, consequently, inhibited plant growth [7,8,9]. Investigating structural changes in roots and leaves is important for a better understanding of metal accumulation and tolerance mechanisms. Excessive metal concentrations have been shown to accelerate maturation of cell walls in the root exodermis and endodermis [9,10]. By disrupting hormonal balance, metals also modify the cell shape and organization, including reductions in the size of mesophyll cells, epidermal cells, and aerenchyma. Metal toxicity has also been shown to decrease the area of vascular bundles and enlarge the size of intercellular spaces in parenchyma tissue [9,11,12].

Leaves, as the primary photosynthetic organs, provide the energy necessary to sustain physiological processes in the entire plant. Protecting photosynthetically active tissues from the harmful effects of metals is therefore crucial. It is also essential to study and understand the alterations in leaf morphology, anatomy, and physiological processes induced by metal toxicity.

Plants have developed several defense mechanisms in response to metal exposure, including metal ion detoxification in the cytoplasm, sequestration in the vacuole, activation of the antioxidative system, and repair of cellular damages [2,4]. The best-known metal-chelating ligands include phytochelatins, organic acids, and amino acids, which reduce free ion concentrations in the cytoplasm and facilitate their sequestration into the vacuole, preventing thus metal interference with essential metabolic processes. Metals present in plant tissues may also be immobilized within carbon-calcium inclusions. The most common forms are calcium oxalate (CaOx) crystals or calcium carbonate (CaCO_3_) cystoliths [13].

Different CaOx crystal morphotypes are known, including raphides, druses, prismatic crystals, and styloids. Raphides are long, needle-shaped structures; druses are star-like aggregates; prismatic crystals are box-like shape; and styloids resemble elongated cuboids or columns [14]. Cystoliths, in turn, are amorphous structures resembling a spindle or a cluster of grapes in which a cellulosic stalk, impregnated with calcium carbonate and a small admixture of silica, is connected to the cell wall as if suspended from it [15]. Cystoliths are usually located in photosynthetic organs, predominantly within epidermal or mesophyll cells known as lithocysts, whereas CaOx crystals may be found in almost any organ or tissue of the plant. Moreover, unlike CaOx crystals, which are widespread across the plant kingdom, cystoliths are restricted to only a few families of dicotyledonous angiosperms [16,17]. The main functions of carbon-calcium inclusions include the regulation, sequestration, or excretion of calcium ions and the maintenance of ionic homeostasis [13,18]. They may also contribute to heavy metal detoxification by sequestering metals away from vital cellular functions [13,18,19]. CaOx crystals deposited near phloem cells may help regulate cytosolic Ca^2+^ levels [20]. In addition, they are involved in plant defense against pathogens [21]. Raphides can protect against herbivores, while other forms of crystals can deter caterpillar larvae [22]. Interestingly, CaOx crystals in leaves can serve as carbon reservoirs, as their decomposition releases CO_2_ that can be reused in photosynthesis [13,23]. The breakdown of CaOx also produces hydrogen peroxide (H_2_O_2_), which may trigger programmed cell death (PCD) and promote cell wall lignification. During PCD, aerenchyma can form, and crystal deposits are frequently observed along the edges of newly formed aerenchymatous cells [24,25]. Some authors refer to these air spaces as fusoid cells. Fusoid cells develop from the ground meristem or parenchyma and, in young leaves, are living cells likely involved in starch synthesis and storage. In mature leaves, they differentiate, enlarge, collapse and die, forming large intercellular gas spaces, while their exact function remains unclear [26].

Although metal-polluted habitats are generally unfavorable for plant growth and development, some species have successfully adapted to these challenging environments. In Poland, the Olkusz and Upper Silesia regions (Silesia-Cracow Uplands) include areas with significantly elevated soil metal concentrations. Industrial waste deposits generated by Zn and Pb ore mining and smelting have accumulated extensively in these regions [27,28]. One such metal-rich waste deposit, over 130 years old, is located in Bolesław near Olkusz. Metal concentrations in the top layer of the waste-heap substrate reach up to 164,960 mg kg^−1^ Zn, 9190 mg kg^−1^ Pb, and 1464 mg kg^−1^ Cd [28]. Plants colonizing these waste heaps are additionally exposed to drought, deficiencies in essential minerals, intense solar radiation, and strong winds [28,29].

One of the dominant plant species inhabiting the waste heap in Bolesław is *Dianthus carthusianorum* L. (Caryophyllaceae). This species is a pseudo-metallophyte, occurring in both unpolluted and metal-polluted habitats. In the latter, microevolutionary processes have led to the emergence of a metal-tolerant calamine ecotype [30]. *D. carthusianorum* is of particular interest because it can be employed in vegetation restoration and phytostabilisation of metalliferous mine wastes [31,32]. The metallicolous (M) ecotype differs markedly from the non-metallicolous (NM) one in terms of morphology, physiology, and tolerance to Zn, Pb, and Cd [30,33,34].

Numerous studies have aimed to elucidate the mechanisms underlying the adaptation of *D. carthusianorum* to metalliferous environments. Physiological traits such as the accumulation of proline, anthocyanins, photosynthetic pigments, phenolic compounds, phytochelatins, and organic acids have been compared between metallicolous and non-metallicolous ecotypes [30,33,34,35,36]. Significant differences in physiological responses to metals were also observed depending on the growth medium, with metal-polluted soil representing chronic stress and hydroponic solutions supplemented with metals representing acute stress [34]. However, to the best of our knowledge, metal-induced alterations in anatomical structure have not yet been thoroughly investigated in this species.

The aim of this study was to assess the effects of toxic concentrations of Cd, Pb, and Zn on the morphology and anatomy of leaves in metallicolous and non-metallicolous ecotypes of *D. carthusianorum* under chronic and acute metal stress conditions. The concentrations of metals used in the hydroponic experiments were selected based on our previous studies to represent exposure levels that induced clear shoot growth reduction in the NM ecotype while not significantly affecting the M ecotype. The presence, types, and abundance of carbon-calcium inclusions, as well as oxalate concentrations, were also investigated. We hypothesized that metal-induced changes in anatomical structure or the accumulation of CaOx would provide insights into the mechanisms underlying the enhanced metal tolerance of *D. carthusianorum* plants originating from the metalliferous waste heap.

## 2. Results and Discussion

### 2.1. Morphological Studies

**Figure 1:**
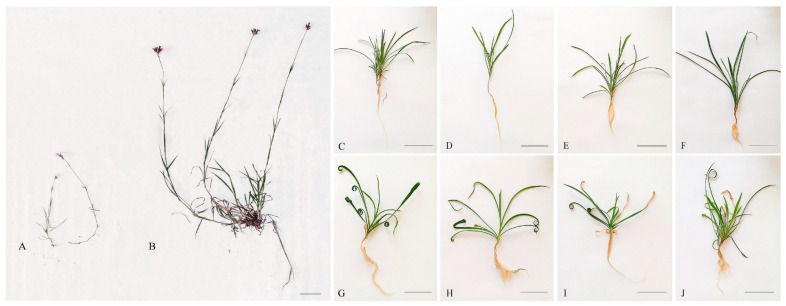
Morphology of *Dianthus carthusianorum* plants. (**A**) Metallicolous ecotype (M) as taken from the field site in Bolesław; (**B**) Non-metallicolous ecotype (NM) as taken from the field site in Pliszczyn. Plants cultivated hydroponically for 14 days in (**C**,**G)** control medium, (**D**,**H**) 50 μM Cd, (**E**,**I**) 30 μM Pb, or (**F**,**J**) 500 μM Zn: (**C**–**F**) M ecotype; (**G**–**J**) NM ecotype. Scale bar = 5 cm.

*Dianthus carthusianorum* forms a single, erect, rigid inflorescence and bears narrowly oblong leaves (Figure 1). Plants collected from the unpolluted site in Pliszczyn were noticeably larger than those from the metal-polluted site in Bolesław (Figure 1A,B). The average fresh weight of leaves in the M ecotype was approximately 2.8-fold lower per plant compared with the NM ecotype, and the leaves were significantly shorter (Table 1). The inflorescence length of the M ecotype averaged 25.53 cm (SD = 6.17), whereas that of the NM ecotype reached 48.15 cm (SD = 13.04). However, when grown under controlled experimental conditions, neither ecotype produced inflorescences during the study period. In the NM plants cultivated under these conditions, leaf tips exhibited curling (Figure 1G–J), a phenomenon not observed in the leaves of the M ecotype. The fresh weight and dimensions (length, width) of NM leaves grown on the control nutrient medium (without metal supplementation) were comparable to those of the M plants, although the dry weight was higher (Table 1).

The dwarfed shoot form observed in ecotypes inhabiting metalliferous habitats is not exceptional. Similar reductions in shoot size have been reported in many plant species, including *Silene vulgaris* [37], *Cardaminopsis halleri* [38], *Cardaminopsis arenosa* [39], and *D. carthusianorum* [30,33]. Such dwarfism may result not only from metal toxicity but also from the nutrient scarcity and xerothermic conditions commonly occurring at metalliferous sites. Nevertheless, it is generally regarded as an evolved trait that enhances survival in high-metal soils [40]. This phenotype reflects an energy trade-off, whereby resources are diverted from biomass production (growth) toward mechanisms of metal tolerance and detoxification. Activation of defense pathways often leads to growth suppression, even in the absence of visible tissue damage, due to competition for limited metabolic and energetic resources [2]. The present study confirmed that the M ecotype is characterized by increased, genetically established tolerance to Zn, Pb, and Cd, as evidenced by a less pronounced reduction in shoot growth parameters following metal exposure in hydroponic cultures [34].

The addition of Cd, Pb, or Zn salts to the nutrient medium under controlled conditions significantly reduced the fresh weight of the NM ecotype leaves, by approximately twofold in the case of Cd and Pb, and threefold following the application of 500 µM Zn. A significant reduction in leaf length, but not width or area, was also observed (Table 1). In contrast, metal toxicity symptoms were considerably less pronounced in the M ecotype. A significant decrease in leaf fresh weight was found only in Zn-treated plants. Although leaf length was also decreased in response to all three metals, the extent of the reduction was smaller than in NM plants. Dry leaf weight corresponded to the pattern observed for fresh leaf weight following metal treatment, indicating that the decrease in fresh weight was not attributable to altered water balance. Indeed, leaf water content, both in field-grown and controlled-condition plants, remained relatively stable at approximately 88–93% and was unaffected by metal exposure (Table 1).

Growth reduction is one of the most evident symptoms of metal toxicity in plants [41]. It was also observed in our previous studies on the effects of Cd, Pb, and Zn on *D. carthusianorum* [33,34,35]. In analogous experiments, 50 μM Cd and 30 μM Pb reduced shoot fresh weight by 38 and 49%, respectively, in the NM ecotype, whereas no effect on shoot biomass was recorded in the M ecotype [35,36]. Similarly, 1000 μM Zn caused a 35% reduction in the fresh weight of the NM shoots, while no negative effect was observed in the M ecotype [34]. Earlier studies showed no significant differences in dry weight or water content between control and Cd-treated plants of both ecotypes [36]; however, to the best of our knowledge, dry weight and other leaf morphometric parameters following Pb or Zn treatments have not yet been investigated in this species. Other studies have demonstrated that disturbance of water balance is often an early metal stress-induced response in many plant species [42]. Such disturbances may result from inhibited water uptake by roots as well as impaired short- and long-distance water transport. The two ecotypes of *D. carthusianorum* are adapted to xerothermic environments and therefore most likely possess inherited traits enabling them to maintain stable water balance even under metal stress.

Interestingly, our previous studies revealed that leaves of the M ecotype accumulated higher concentrations of Zn (appr. 3000 mg kg^−1^) and Pb (appr. 370 mg kg^−1^), while Cd concentrations were similar, not statistically different, (appr. 680 mg kg^−1^), compared with the NM ecotype (appr. 2000, 210, and 550 mg kg^−1^, respectively) when cultivated hydroponically under analogous experimental conditions [34,35,36]. This combination of relatively high metal accumulation and only a mild growth reduction suggests that the M ecotype possess more efficient mechanisms of metal detoxification and immobilization than the NM ecotype. Notably, these differences could not be attributed to variations in the synthesis of metal-complexing phytochelatins, organic acids, or proline [34,35,36]. Consequently, the next step of our study focused on leaf anatomy, aiming to determine whether the M ecotype is also more resistant to tissue and cellular damage and to identify anatomical traits that may contribute to its enhanced metal tolerance.

### 2.2. Anatomical and Histochemical Study

**Figure 2:**
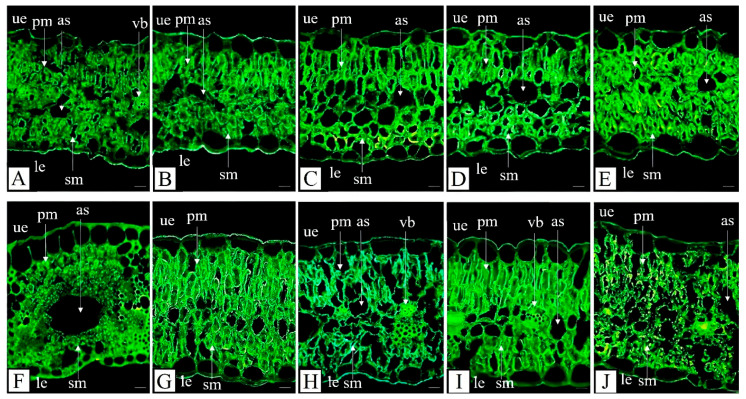
Cross-sections of *Dianthus carthusianorum* leaves. Non-metallicolous ecotype (NM) from the field site in Pliszczyn (**A**) and hydroponically cultivated for 14 days in control medium (**B**) or in medium supplemented with 50 μM Cd (**C**), 30 μM Pb (**D**), or 500 μM Zn (**E**). Metallicolous ecotype (M) from the field site in Bolesław (**F**) and hydroponically cultivated for 14 days in control medium (**G**) or in medium supplemented with 50 μM Cd (**H**), 30 μM Pb (**I**), or 500 μM Zn (**J**). Sections were stained with auramine and observed under fluorescence microscopy. Abbreviations: as—air space, le—lower epidermis, pm—palisade mesophyll, sm—spongy mesophyll, ue—upper epidermis, and vb—vascular bundle. Scale bar = 40 µm.

Analysis of leaf cross-sections provided insights into the tissue organization and cell structure. Leaf blade thickness was similar in both ecotypes and was not affected by metal treatments (Figure 2A–J, Table 2). Likewise, the continuity of both the upper and lower epidermis was preserved, and the epidermal cell size remained consistent across all plants. However, exposure to metals in hydroponically grown plants of both ecotypes induced changes in mesophyll thickness and cell arrangement. Disruption of the mesophyll structure was observed, manifested as reductions in cell numbers or sizes and the formation of intercellular spaces. An exception was the NM ecotype exposed to excess Zn, where mesophyll cells appeared tightly adhered to one another (Figure 2E). Anatomical measurements indicated that palisade mesophyll thickness remained constant in both ecotypes, regardless of growth conditions or metal treatments. In contrast, thickness spongy mesophyll thickness slightly increased in the M ecotype following Cd and Zn exposure, whereas it slightly decreased in the NM ecotype in response to Pb treatment (Table 2).

Numerous anatomical studies on metal exposed plants have reported inconsistent and sometimes contradictory effects on leaf blade and parenchyma cell thickness [9]. For instance, Vaculik et al. [43] reported decreased leaf thickness in Cd-treated maize, whereas Tóth et al. [44] observed that the lamina thickness of white willow increased following metal exposure, which was attributed to expansion of the spongy parenchyma, similar to our observations. Comparable results were reported for *Brachiaria decumbens* Stapf. cv. Basilisk and *Panicum maximum* Jacq. cv. Massai exposed to Cd [45], and for *Peltophorum dubium* (Spreng.) Taub. treated with Pb [46]. However, many species exhibited reduced spongy mesophyll thickness and cellular damage following Cd exposure [47], like in our NM plants exposed to Pb. These variable results may be due to different plant species examined and their inherent metal tolerance, as well as variations in treatment methods, metal concentrations, exposure duration, plant age, and other factors.

**Figure 3:**
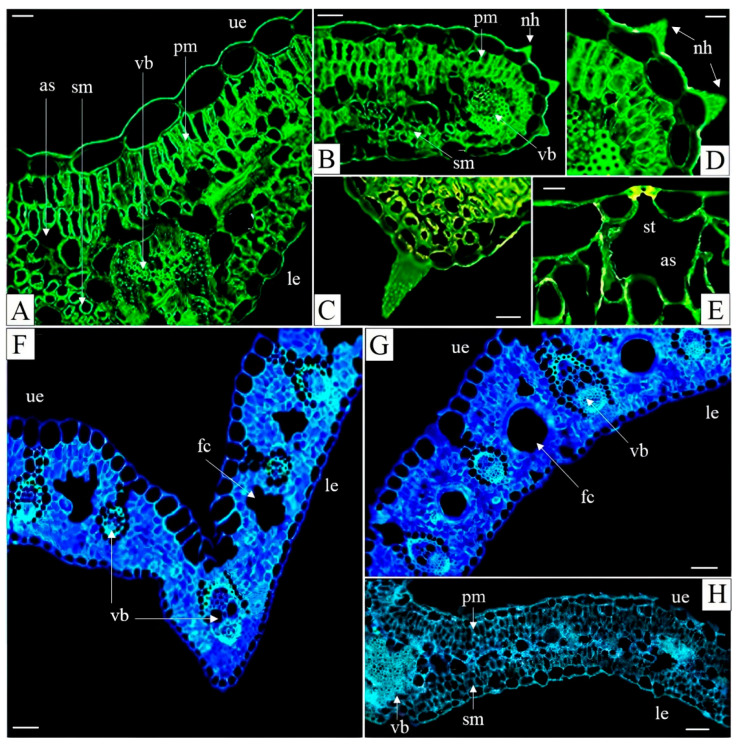
Cross-sections of *Dianthus carthusianorum* leaves. Metallicolous ecotype (M) hydroponically cultivated for 14 days in medium with 30 μM Pb (**A**–**E**,**H**) and from the field site in Bolesław (**F**,**G**). Sections were stained with auramine (**A**–**E**), calcofluor (**F**,**G**), or aniline blue (**H**) and observed using fluorescence microscopy. Abbreviations: as—air spaces, fc—fusoid-like cell, le—lower epidermis, nh—non-glandular hair, pm—palisade mesophyll, sm—spongy mesophyll, st—stomata, ue—upper epidermis, and vb—vascular bundle. Scale bars: (**A**) = 20 µm; (**B**,**F**,**G**) = 40 µm; (**C**,**D**,**E**) = 10 µm; (**H**) = 100 µm.

In our study, an increase in the area of intercellular air spaces was observed in leaves of the M ecotype treated with Pb and Zn, and in leaves of the NM ecotype in response to all three metals (Figure 2 and Figure 3A,B; Table 2). The expansion of air spaces was significantly greater in the NM ecotype following metal exposure. The largest air spaces areas were found in leaves of NM plants exposed to Zn and Pb (appr. 2.8 times larger than in control plants), while Cd exposure also resulted in a substantial increase (appr. 2.2 times larger than in control) (Table 2).

Interestingly, large spaces resembling morphologically fusoid cells were observed in the leaves of *D. carthusianorum* from the metal-polluted site (Figure 3F,G). Fusoid cells are large, specialized cells, typically found in the leaves of Poaceae, as well as in several species belonging to Flagellariaceae and Joinvilleaceae families [26]. To date, they have not been described in species outside these families, including Caryophyllaceae, making our observation the first report of their potential presence in this family. The function of fusoid cells is not yet fully understood, but their position near vascular tissues suggest that they may facilitate symplastic water transport from vascular bundles to mesophyll cells. It was proposed that fusoid cells may function in water storage and protection against excessive water loss under high temperature or drought [48]. This hypothesis is particularly interesting, given that the metallicolous ecotype of *D. carthusianorum* grows on dry substrates with high insolation. The spaces formed by fusoid cells may also contribute to CO_2_ capture in the leaf [49] or enhance light interception in shaded leaves [50], thereby improving photosynthetic efficiency.

**Figure 4:**
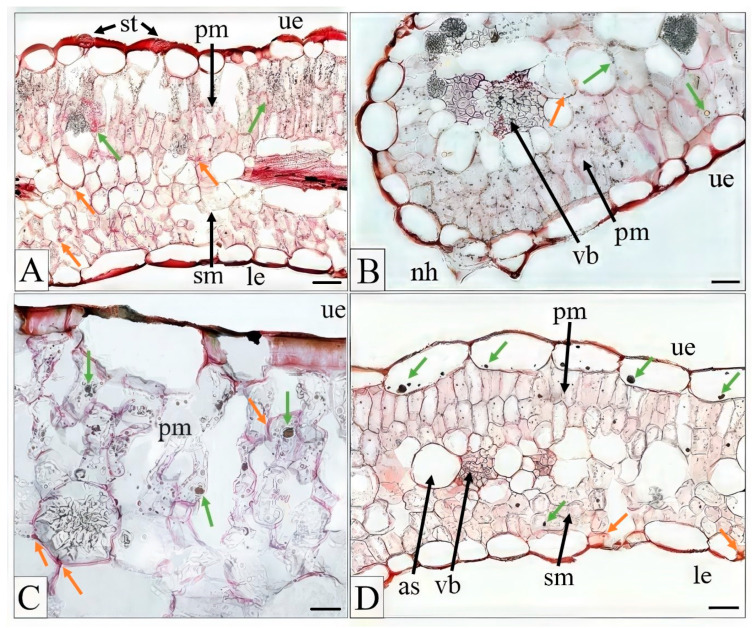
Cross-sections of *Dianthus carthusianorum* leaves showing accumulation of polysaccharides and tannins. Metallicolous ecotype (M) hydroponically cultivated for 14 days in nutrient medium supplemented with Cd (**A**,**C**) or Pb (**B**,**D**). Sections were stained with 10% FeCl_3_ in methanol combined with ruthenium red and Schiff’s reagent and observed using light microscopy. Orange arrows indicate polysaccharides; green arrows indicate tannins. Abbreviations: as—air space, le—lower epidermis, nh—non-glandular hair, pm—palisade mesophyll, sm—spongy mesophyll, st—stomata, ue—upper epidermis, and vb—vascular bundle. Scale bars: (**A**,**B**,**D**) = 20 µm; (**C**) = 10 µm.

Non-glandular trichomes—small, delicate hair-like structures composed of a few cells with sharp tips—were observed on the margins of all leaves (Figure 3B–D and Figure 4B). In general, the leaf blades were hairless or bore very few hairs, suggesting that these trichomes are unlikely to play a significant role in protection against excessive water loss of herbivory. Stomata were present on both the dorsal (adaxial) and ventral (abaxial) leaf surfaces (Figure 4A,D), consistent with observations in other *Dianthus* species, e.g., [51].

**Figure 5:**
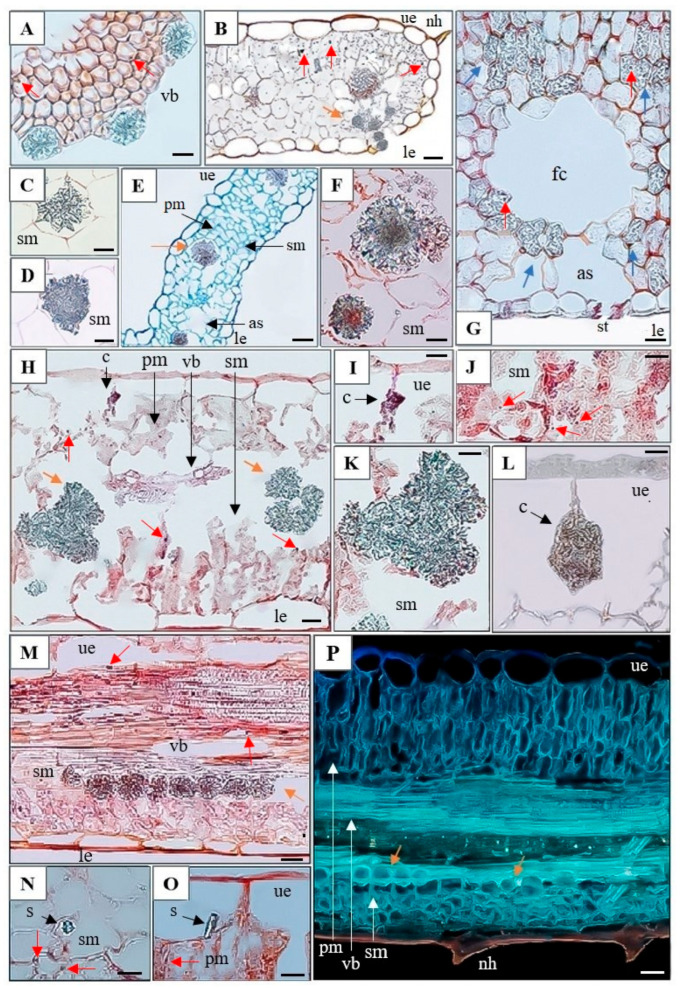
Cross-sections of *Dianthus carthusianorum* leaves showing calcium inclusions. Metallicolous ecotype (M) from Bolesław hydroponically cultivated for 14 days in medium with 30 μM Pb (**A**,**D**,**M**–**O**) and from the field site (**G**); non-metallicolous ecotype (NM) from Pliszczyn hydroponically cultivated for 14 days in medium with 50 μM Cd (**E**), 30 μM Pb (**B**,**C**,**F**,**H**–**L**), or 500 μM Zn (**P**). Sections were stained with ruthenium red (**A**–**D**,**F**,**G**,**M**,**N**,**O**), toluidine blue (**E**), Sudan IV (**H**–**L**), or aniline blue (**P**) and observed using light microscopy (**A**–**O**) or fluorescence microscopy (**P**). Orange arrows indicate calcium oxalate crystals; red arrows indicate oil droplets; blue arrows indicate irregularly shaped crystals in M ecotype from the field. Abbreviations: as—air space, c—cystolith, fc—fusoid-like cell, le—lower epidermis, nh—non-glandular hair, pm—palisade mesophyll, sm—spongy mesophyll, s—styloid, st—stomata, ue—upper epidermis, and vb—vascular bundle. Scale bars: (**A**,**F**,**I**,**J**,**K**,**L**,**N**,**O**) = 10 µm; (**B**) = 40 µm; (**C**,**D**,**G**,**H**,**M**,**P**) = 20 µm; (**E**) = 100 µm.

Shiff’s reagent, used to detect polysaccharides, and FeCl_3_, used to visualize tannins, revealed their presence in all leaf cross-sections from every treatment, although these compounds were virtually more abundant in the M ecotype. Representative images illustrating their presence are shown in Figure 4A–D. Polysaccharides were primarily accumulated in mesophyll cells, whereas tannins were observed both in palisade mesophyll and upper epidermis cells. Similarly, oil droplets were detected in leaf cross-sections of all examined plants (Figure 5).

Numerous calcium inclusions were observed in the leaves of both ecotypes (M and NM), whether collected from their natural habitats or grown hydroponically in media supplemented with Cd, Pb, and Zn (Figure 5). Large druses were typically located in the central parts of the leaves (e.g., Figure 5E,F,H,K), while smaller CaOx crystals were aligned along the vascular tissue margins (e.g., Figure 5A,B,M,P).

The highest number of druses was observed in the leaves of hydroponically grown M plants exposed to Pb (Table 3). Small druses adjacent to vascular tissues were particularly abundant in leaves of the M ecotype exposed to excess Zn (Figure 5), although their overall abundance was comparable to that in Pb- and Cd-treated plants (Table 3). An increase in the area occupied by druses was evident in metal-treated plant of both ecotypes (Table 3). Leaves of field grown M plants contained numerous irregularly shaped crystals embedded at the edges of fusoid-like cells (Figure 5G, Table 3). In leaves of hydroponically grown M plants exposed to Pb, two types of CaOx crystals were observed: styloids, consisting of single elongated crystals with pointed ends (Figure 5N,O), and druses, occurring individually or in clusters (e.g., three clustered druses are visible in Figure 5H,K).

CaOx crystals have been extensively studied due to their widespread occurrence in the plant kingdom and their multiple and diverse biological functions and ecological significance [13,14]. They are formed in the vacuoles of specialized cells called idioblasts from endogenously synthesized oxalic acid and calcium absorbed from the environment [52]. CaOx crystals are primarily involved in calcium storage, transport, and homeostasis; however, they also contribute to defense against pathogens and herbivores, support photosynthesis under harsh environmental conditions, and participate in the management of excess ions [20,21,22,53]. These functions are particularly advantageous for plants growing in metalliferous waste deposits or otherwise exposed to metals, as in the present study. For instance, decomposition of these crystals can supply additional carbon for photosynthesis, representing an adaptative strategy for growth in dry environments by conserving water and reducing carbon loss to the atmosphere [23].

Under intense insolation, CaOx crystals can reflect excess radiation, providing partial protection to chloroplasts against photodamage and reducing photoinhibition [54]. The presence of numerous crystals near vascular tissues suggests a role in calcium transport, facilitating mobilization and redistribution under Ca-poor soil conditions [55]. Importantly, in the context of our study, CaOx crystals may also contribute to trace metal detoxification. Weber et al. [56], using various chemical analyzes including high-resolution synchrotron X-ray powder diffraction, energy-dispersive X-ray spectroscopy (EDX), inductively coupled plasma atomic emission spectroscopy, and transmission electron microscopy (TEM), demonstrated that Ca^2+^ can be substituted by metals when crystals form in the presence of Pb^2+^, Cd^2+^ or Zn^2+^. Large quantities of crystals were observed in the leaves of the M ecotype from the waste heap, as well as in leaves of both M and NM plants grown hydroponically with added metals. This suggests that metals can be co-precipitated within CaOx crystals as a mechanism of detoxification. Similar observations were reported in cacao trees exposed to Cd [57]. However, Faheed et al. [52] found that the addition of metals (Cd, Pb, Zn, Cu) to the nutrient medium decreased the number of crystals in leaves of *Corchorus olitorius* L. and *Malva parviflora* L., and found no evidence of metal inclusion in CaOx crystals by EDX, indicating species-specific differences.

In addition to CaOx crystals, cystoliths were observed in the leaves of the NM ecotype exposed to Pb. The process of cystolith formation is illustrated in Figure 5H,I, with a mature cystolith in Figure 5L. To our knowledge, this represents the first report of cystoliths in a species of the Caryophyllaceae family. To date, cystoliths have been well documented in eight families: Acanthaceae, Boraginaceae, Cannabaceae, Cucurbitaceae, Hernandiaceae, Moraceae, Opiliaceae, and Urticaceae. They have also been tentatively reported in some species of Asteraceae, Begoniaceae, Brassicaceae, Burseraceae, Campanulaceae, Cistaceae, Convolvulaceae, Linderniaceae, Loasaceae, Orobanchaceae, Rubiaceae, Salicaceae, Ulmaceae, and Verbenaceae, although further confirmation and documentation are needed in these families [16].

Like CaOx crystals, cystoliths are believed to act as reservoirs or regulators of calcium and may support photosynthesis, either by scattering light to distribute it more evenly within the leaf or by supplying CO_2_ to mesophyll cells [15,16]. Their role in enhancing photosynthesis is supported by their exclusive localization in abaxial and adaxial epidermal cells as well as in mesophyll cells, and may be linked to specific leaf anatomy and unfavorable environmental conditions, such as low light intensity or the need to store CO_2_ under water deficit conditions when stomata are closed [58]. Whether cystoliths also contribute to metal detoxification remains to be elucidated.

### 2.3. Insoluble and Soluble Oxalate Concentrations

**Figure 6:**
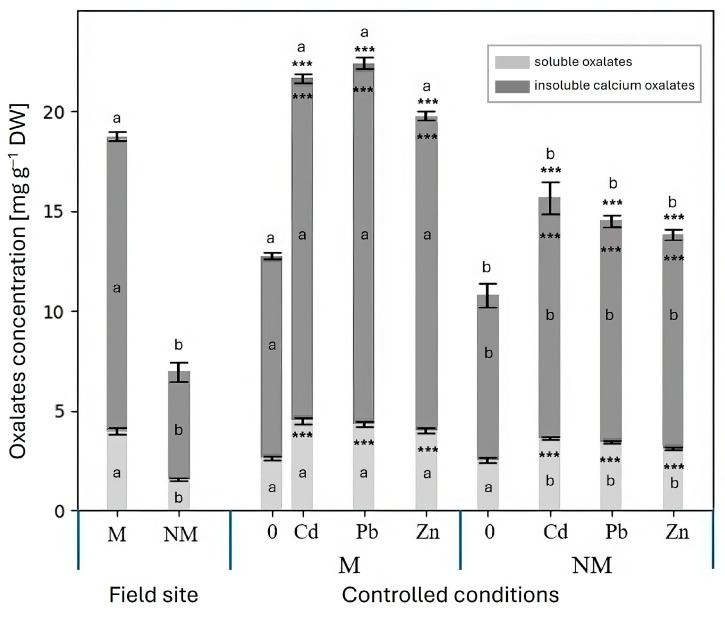
Concentrations of soluble and insoluble oxalates in the leaves of *Dianthus carthusianorum* plants from the metallicolous (M) and non-metallicolous (NM) ecotypes taken from the field sites or hydroponically grown under controlled conditions for 14 days in control medium (0) or in medium supplemented with 50 μM Cd, 30 μM Pb, or 500 μM Zn. Asterisks indicate statistically significant differences between metal-treated plants and the respective control within the same ecotype at *** *p* < 0.001. Two ecotypes (M and NM) were compared under the same experimental conditions using letters; values followed by the same letters do not differ significantly (n = 10).

Two forms of oxalates ere determined and quantified in the leaves of studied plants: insoluble oxalates (i.e., calcium oxalate, observed as crystals in leaf cross-sections, as described above) and soluble oxalates (e.g., sodium and potassium oxalate) (Figure 6), with the latter being by far the dominant form. Remarkably, under field conditions, leaves of the M ecotype contained nearly twice the concentrations of both soluble and insoluble oxalates compared with the NM ecotype. This strongly suggests a role for oxalates in mitigating metal stress, as excess metal ions may be detoxified through precipitation as insoluble, metabolically inactive CaOx crystals.

Interestingly, concentrations of insoluble CaOx were also higher in leaves of the M ecotype grown under controlled conditions in the absence of metal supplementation. This suggests a genetically established adaptation of the M ecotype to elevated metal availability, along with other adaptive traits such as increased levels of organic acids, particularly malate and citrate [34]. Addition of Cd, Pb, or excess Zn to the nutrient solution further increased concentrations of both soluble and insoluble oxalates in both ecotypes, with consistently higher levels observed in the M ecotype. These results reinforce the important role of CaOx in metal tolerance and in mitigating metal stress, particularly in the M ecotype.

**Figure 7:**
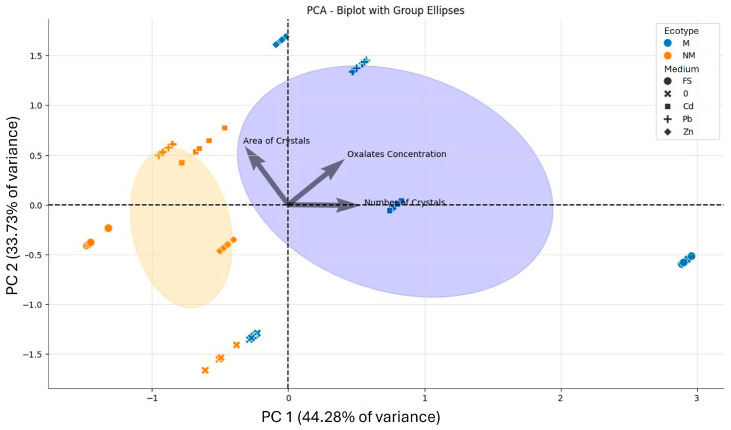
Principal Component Analysis of oxalates concentration and CaOx crystal number and area in the leaves of *Dianthus carthusianorum* plants from the metallicolous (M) and non-metallicolous (NM) ecotypes taken from the field sites (FS) or hydroponically grown under controlled conditions for 14 days in control medium (0) or in medium supplemented with 50 μM Cd, 30 μM Pb, or 500 μM Zn. The purple and yellow areas refer to M and NM ecotypes, respectively.

Figure 7 further illustrates the differences in oxalate content between leaves of the M and NM ecotypes. A biplot combining oxalate concentration with the area and number of CaOx crystals observed in leaf cross-sections explained 78% of the total variance in the dataset. The first principal component accounted for 44.28% of the variance and separated the M and NM ecotypes, as well as experimental variants, based on the number of CaOx crystals and oxalate concentrations. The second component explained 33.73% of the variance and was primarily associated with the area of crystals. The distinction between the M and NM ecotypes was particularly evident in field-grown plant, with leaves from the metalliferous waste deposit showing the largest accumulation of CaOx. The PCA also clearly showed that metal application in the hydroponic nutrient medium enhanced oxalate production and crystal deposition, particularly in the M ecotype.

Given the abundant CaOx crystals accumulation visible in leaf sections and the increased oxalate concentrations following metals treatment, especially in the M ecotype, the potential energetic costs of this defense mechanism warrant consideration. Oxalates play dual roles in plants, both in regulating calcium homeostasis and in providing physical defense against herbivores or contributing to metal detoxification [13,14]. However, their synthesis sequester calcium and carbon, potentially affecting nutrient use efficiency and plant growth, representing a trade-off between defense and growth. Indeed, in field-grown plants exposed to chronic metal stress, elevated CaOx levels in the M ecotype coincide with reduced growth relative to the NM ecotype from the reference habitat, suggesting a shift in resources toward defense. In contrast, in hydroponically grown plants exposed to metals, although CaOx accumulation remains higher in the M ecotype, it does not lead to reduced growth, highlighting a balance between this defensive investment and overall resource allocation.

## 3. Materials and Methods

### 3.1. Plant Material and Growth Conditions

**Figure 8:**
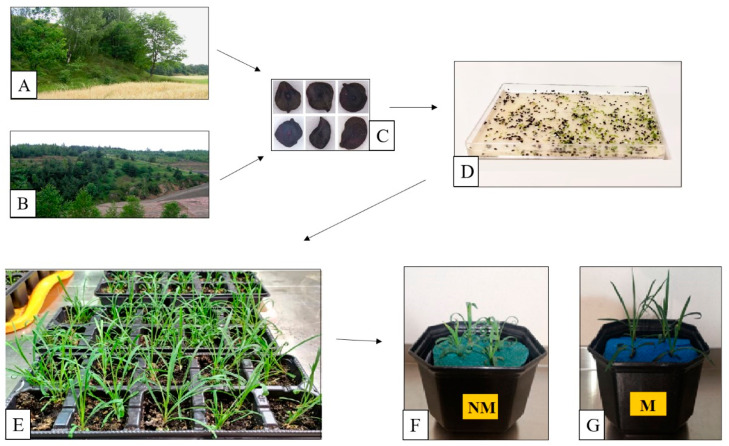
Experimental procedure. Plants of *Dianthus carthusianorum* from non-polluted (**A**) and polluted (metalliferous waste deposit, (**B**)) sites were examined and served as the source of seeds (**C**) for plant cultivation under controlled conditions. Plant cultivation comprised seed germination (**D**), preliminary growth in potting soil (**E**), and hydroponic growth in nutrient solution (**F**,**G**) for exposure to metals. NM—non-metallicolous ecotype; M—metallicolous ecotype.

Two ecotypes of *Dianthus carthusianorum* L. (Caryophyllaceae) were investigated in this study. The reference non-metallicolous ecotype (NM) originated from a non-polluted site in Pliszczyn near Lublin, eastern Poland (51°18′ N, 23°41′ E) (Figure 8A). The metallicolous ecotype (M) originated from a waste heap left after mining and processing of Zn-Pb ores located in Bolesław near Olkusz, southern Poland (50°17′ N, 19°29′ E) (Figure 8B). The characteristics of the sampling sites was described previously [30].

Turf fragments with *D. carthusianorum* plants were collected from the field sites (Figure 8A,B) and transported to the laboratory to obtain material for analyses. The seeds (Figure 8C), collected in bulk from different mother plants inhabiting both sites, were stratified for several weeks at 4 °C and then germinated on moist filter paper in covered plastic dishes (Figure 8D). Five- to seven-day-old seedlings were transplanted into sowing trays (Figure 8E) filled with commercially available potting soil that had been double autoclaved. Seven weeks after sowing, the plants were transferred into polyethylene pots containing 0.5 L of modified Hoagland solution (pH ≈ 5.6) [59], which contained 1 mM NH_4_H_2_PO_4_, 6 mM KNO_3_, 4 mM Ca(NO_3_)_2_, 2 mM MgSO_4_, 46 μM H_3_BO_3_, 9 μM MnCl_2_, 0.76 μM ZnSO_4_, 0.32 μM CuSO_4_, 0.11 μM H_2_MoO_4_, and 85 μM Fe supplied as Fe (III) citrate (all chemicals from POCh, Gliwice, Poland). During the transfer, roots were carefully washed to remove all adhering soil particles. Two plants were cultivated in each pot (Figure 8F,G). Plants were acclimated for two days under hydroponic conditions, after which the nutrient solution was renewed. Subsequently, plants were exposed either to untreated solution (control) or to solution supplemented with 50 μM Cd, 30 μM Pb, or 500 μM Zn, supplied as Cd(NO_3_)_2_ × 4 H_2_O, Pb(NO_3_)_2_ (both purchased from Sigma-Aldrich, St. Louis, MO, USA), or ZnSO_4_ × 7 H_2_O (POCh, Gliwice, Poland), respectively. The concentrations of metals were selected based on our previous studies [34,35,36], which showed that these levels reduced shoot biomass of the NM ecotype by 35–49% while having no significant effect on shoot biomass of the M ecotype.

In total, plants remained in the hydroponic solution for 16 days, including the acclimation period. The solutions were continuously aerated. After seven days from metal addition all solutions, including controls, were renewed with metals added at the same concentrations. Plants were analyzed after 14 days of metal exposure. Plants were grown under controlled conditions in a growth chamber at 24/18 °C (day/night), at a 16-h photoperiod, photosynthetic active radiation of 150 μmol m^−2^ s^−1^, and approximately 60% relative humidity.

### 3.2. Morphological Parameters and Water Content

The plants of both ecotypes, obtained from the field sites as well as from each treatment in the hydroponic culture, were analyzed for the following morphological parameters: fresh weight (FW), dry weight (DW), length, width, and area of leaves. To determine the dry weight, leaf material was dried at 70 °C until constant weight. Water content was calculated using the formula: (FW–DW)/FW × 100% and expressed as a percentage of the fresh weight. Leaf area measurements were performed using the Easy Leaf Area application (https://play.google.com/store/apps/details?id=com.heaslon.EasyLeafArea&hl=en_US&pli=1; accessed on 21 October 2024). Ten plants from each experimental variant were used for the analyses.

### 3.3. Preparation of Leaf Samples for Anatomical and Histological Analyses

For anatomical analyses, 3–4 mm fragments spanning the whole leaf width were collected from fully expanded 2–4 leaves in the middle part of the rosette. The 2–4 oldest leaves, located at the base of the rosette, and four youngest leaves, located in its center, were excluded. Each sample was taken 0.5 cm below the leaf apex and cut perpendicular to the leaf length toward the leaf base. All chemicals used for sample preparation and dyes were purchased from Sigma-Aldrich (St. Louis, MO, USA). Samples were fixed overnight in Carnoy’s solution (a mixture of 96% ethanol and 80% glacial acetic acid) and then vented using a vacuum pump (10 cycles × 15 min at 0.8 atm). After fixation, the samples were rinsed for 15 min in 96% ethanol and subsequently in anhydrous ethyl alcohol (99.8%) (3 × 15 min). The dehydrated material was saturated with mixtures of acetone and anhydrous ethyl alcohol in ratios of 1:3, 1:1, and 3:1 (30 min each), followed by three washes in pure dehydrated acetone (3 × 15 min). Next, the samples were saturated with paraffin in a laboratory hothouse at 37 °C for 12 h and then 58 °C for five days, with paraffin replaced twice daily. Finally, they were embedded in paraffin blocks. Cross-sections for anatomical analyses were performed on a total of 10 different leaves.

The solidified blocks were sectioned into 5 µm slices using a Leica RM 2125 RTS rotary microtome (Leica Microsystems, Wetzlar, Germany). The sections were mounted on slides using Haupt’s mastic and 10% ethanol. Paraffin was removed from the sections using xylene, followed by a series of decreasing alcohol concentrations for rehydration. The deparaffinized sections were stained with various dyes (Table 4). After staining, a drop of glycerin was applied on the sections (Canada balsam was used only after staining with Sudan IV or ruthenium red), and the samples were covered with a coverslip. For the observation of carbon–calcium inclusions, the slides were stained with ruthenium red, toluidine blue, and Sudan IV.

### 3.4. Anatomical and Histological Analyses

A Nikon OPTIPHOT-2 microscope (Nikon Corporation, Tokyo, Japan) was used for microscopic observations. Images were captured using a Nikon Coolpix 4500 camera (Nikon Corporation). Selected images were processed with Adobe Photoshop 7.0 (Adobe Systems Inc., San Jose, CA, USA). Leaf cross-sections were analyzed to determine the thickness of the leaf blade, upper and lower cuticle, upper and lower epidermis, palisade and spongy mesophyll, as well as the proportion of intercellular air spaces. Moreover, the presence, type, number, and area of calcium-carbon inclusions, including calcium oxalate crystals and cystoliths, was analyzed. Measurements were performed on calibrated microscopic images using Image-Pro Plus software, version 5.1 (Media Cybernetics, Silver Spring, MD, USA), ensuring measurement accuracy and consistency across experimental variants. The presence of oil droplets, polysaccharides, and tannins was also recorded. For each experimental variant, a minimum of 10 cross-sections from 10 different leaves were examined.

### 3.5. Determination of Soluble and Insoluble Oxalate Concentrations

In plant tissues, oxalic acid occurs in two forms: soluble oxalates, usually present as salts with monovalent cations such as sodium (Na^+^), potassium (K^+^) and ammonium (NH_4_^+^); and insoluble oxalates, forming salts with divalent ions, including calcium (Ca^2+^), magnesium (Mg^2+^), and iron (Fe^2+^). Analyses of soluble and total oxalates in *D. carthusianorum* leaves were performed using a modified procedure described by Jabłonowski [64] and Mishra et al. [65]. All chemicals were purchased from Sigma-Aldrich (St. Louis, MO, USA), unless otherwise stated.

Crushed dry leaf samples were placed in beakers and extracted either with distilled water (extract A, for soluble oxalates) or with 1 M HCl (POCh, Gliwice, Poland) (extract B, for total oxalates), in each case using a 30:30 (g/mL) ratio (*w*/*v*). The mixtures were boiled for 15 min, after which 200 mL of water was added to each beaker, and the suspensions were left overnight. Both extracts were subsequently filtered through Whatman’s No. 1 filter paper and deproteinized by adding 1.25 mL of phosphotungstic reagent and 1.25 mL of 6 M HCl to extract A, and 2.5 mL of phosphotungstic reagent to 15 mL of extract B (the color changed to blue). The solutions were left for 5 h and then centrifuged at 3000 rpm for 10 min. To the supernatants, 10 mL of 5% ammonia, 2.5 mL of calcium chloride buffer (pH 4.5), and 1.25 mL of acetone were added to precipitate oxalate. The samples were centrifuged again at 3000 rpm for 10 min, the supernatants were carefully removed, and the pellets were washed with 10 mL of 5% acetic acid, followed by another centrifugation under the same conditions. After removing the supernatants, the pellets were dissolved in 2.5 mL of 10% sulfuric acid and heated in a water bath at 95–100 °C for 2 min. The samples were titrated with 0.02 M KMnO_4_. The following assumptions were adopted: 1 mL of 0.02 M KMnO_4_ corresponds to 0.9 mg (COOH) and 10 mL of deproteinized extract corresponds to 1 g of raw material. The calculated concentrations of soluble and insoluble oxalates were expressed as mg g^−1^ DW.

### 3.6. Statistical Analyzes

Statistical analyses were performed using ANOVA in IBM SPSS Statistics software, version 26.0 (IBM Corp., Armonk, NY, USA) to evaluate differences between specific groups. Prior to the analysis, all data were tested for normality with the Shapiro-Wilk test and variance heterogeneity was checked using Levene’s test. Consequently, Student’s *t*-test or the Mann-Whitney *U*-test was applied. Asterisks indicate statistically significant differences between metal-treated plants and the respective controls within each ecotype at the significance levels: * *p* < 0.05; ** *p* < 0.01; *** *p* < 0.001. Comparisons between the two ecotypes (M and NM) under the same experimental conditions were performed; values followed by the same letters do not differ significantly. PCA analysis was conducted in RStudio (version 2022.02.0) using R software (version 4.1.0; R Foundation for Statistical Computing, Vienna, Austria). All measured and calculated parameters were expressed as means ± SD, and the differences were considered significant at *p* < 0.05. 

## 4. Conclusions and Future Directions

The present study provides novel insights into the anatomical and biochemical adaptations of *D. carthusianorum* that enable survival and growth in metalliferous environments. Revisiting our hypothesis—that modifications in anatomical structure and enhanced accumulation of CaOx contribute to the superior metal tolerance of the M ecotype—we provide strong evidence supporting both mechanisms. Our results clearly demonstrate that the M ecotype exhibits markedly higher tolerance to Cd, Pb, and Zn, as reflected not only in less pronounced reductions in leaf growth under metal stress but also substantially elevated concentrations of both soluble and insoluble oxalates. Anatomical analyses further revealed that leaves of the M ecotype sustained less tissue damage under metal exposure compared to the NM ecotype, with smaller expansions of intercellular air spaces and preserved mesophyll structure, indicating enhanced tissue resilience. The consistently higher abundance of CaOx crystals in the M ecotype, independent of growth conditions and regardless of chronic or acute metal stress, indicates that their formation represents a genetically fixed adaptation, contributing to metal detoxification through immobilization of excess ions in a metabolically inert form. Notably, for the first time, cystoliths were detected in a species of the Caryophyllaceae family, although their functional role in *D. carthusianorum* remains to be elucidated, as they were observed only in a single experimental variant. Overall, these findings highlight the complex interplay between anatomical and biochemical mechanisms in conferring metal tolerance, representing a significant advance over previous studies that have primarily focused on physiological traits.

Future research should prioritize a similar detailed analysis of root tissues, which are the first organs exposed to metals and likely to exhibit early signs of toxicity as well as the primary detoxification mechanisms. Integrating root anatomical studies with subcellular metal localization techniques (e.g., micro X-ray fluorescence, µXRF; TEM-EDX) would provide a more comprehensive understanding of detoxification pathways. Moreover, comparative transcriptomic or metabolomic analyses between M and NM ecotypes could elucidate molecular networks regulating oxalate synthesis, CaOx crystal formation, and other tolerance related mechanisms.

Together, such investigations would complement the current leaf-based analyses, offering a holistic view of the adaptations enabling *D. carthusianorum* to thrive in metalliferous environments, and provide a strong foundation for its potential application in phytostabilisation strategies.

## Figures and Tables

**Table 1:** Morphological parameters and water content of leaves of *Dianthus carthusianorum* plants from the metallicolous (M) and non-metallicolous (NM) ecotypes. Plants were collected from field sites or cultivated hydroponically under controlled conditions for 14 days in the control medium (0) or in the medium supplemented with 50 μM Cd, 30 μM Pb, or 500 μM Zn.

	Field Site		Controlled Conditions							
	M	NM	M				NM			
Parameter			0	Cd	Pb	Zn	0	Cd	Pb	Zn
fresh weight of leaves [g]	0.243 b ±0.12	0.685 a ±0.06	0.444 a ±0.16	0.382 a ±0.1	0.338 a ±0.03	0.238 a** ±0.06	0.753 a ±0.26	0.382 a* ±0.13	0.344 a** ±0.03	0.241 a*** ±0.08
dry weight of leaves [g]	0.017 b ±0.01	0.070 a ±0.02	0.027 b ±0.02	0.021 a ±0.01	0.039 a ±0.03	0.027 a** ±0.03	0.085 a ±0.01	0.052 a** ±0.01	0.039 a** ±0.03	0.023 a*** ±0.02
water content [% of fresh weight]	93.00 a ±7.4	89.78 a ±3.5	93,91 a ±5.0	94.50 a ±3.6	88.46 a ±11.2	89,90 a ±9.8	88.7 b ±8.4	86.39 a ±17.8	88.66 a ±9.3	90.46 a ±8.0
leaf length [cm]	5.1 b ±0.94	7.3 a ±1.6	7.6 a ±1.31	6.2 a** ±0.81	6.3 a** ±0.61	5.3 a*** ±0.51	7.3 a ±0.63	4.9 b*** ±0.85	5.2 b*** ±0.75	5.4 a*** ±0.72
leaf width [cm]	0.32 a ±0.08	0.36 a ±0.05	0.27 a ±0.05	0.28 a ±0.04	0.29 a ±0.03	0.29 a ±0.09	0.26 a ±0.05	0.25 a ±0.05	0.27 a ±0.05	0.27 a ±0.05
leaf area [cm^2^]	1.29 a ±0.81	1.33 a ±0.69	1.67 a ±0.55	1.38 a ±0.63	1.27 a ±0.78	1.33 a ±0.84	1.18 a ±0.64	1.34 a ± 0.57	1.40 a ±0.49	1.33 a ±0.57

Asterisks indicate statistically significant differences between metal-treated plants and the respective control within the same ecotype: * *p* < 0.5; ** *p* < 0.01; *** *p* < 0.001. Differences between ecotypes (M and NM) under the same experimental conditions are indicated by letters; values followed by the same letters do not differ significantly (n = 10).

**Table 2:** Anatomical parameters of *Dianthus carthusianorum* leaves from the metallicolous (M) and non-metallicolous (NM) ecotypes. Plants were collected from the field sites or cultivated hydroponically under controlled conditions for 14 days in the control medium (0) or in medium supplemented with 50 μM Cd, 30 μM Pb, or 500 μM Zn.

	Field Sites		Controlled Conditions							
	M	NM	M				NM			
Parameter			0	Cd	Pb	Zn	0	Cd	Pb	Zn
leaf blade thickness [mm]	0.33 a ±0.03	0.32 a ±0.02	0.33 a ±0.03	0.33 a ±0.03	0.33 a ±0.03	0.32 a ±0.01	0.32 a ±0.02	0.32 a ±0.02	0.32 a ±0.03	0.32 a ±0.02
upper cuticle thickness [µm]	3.74 a ±0.45	3.78 a ±0.38	3.47 a ±0.3	3.61 a ±0.71	3.41 a ±0.62	3.66 a ±0.61	3.7 a ±0.58	3.62 a ±0.89	3.51 a ±0.61	3.7 a ±0.8
lower cuticle thickness [µm]	3.74 a ±0.18	3.69 a ±0.25	3.67 a ±0.15	3.61 a ±0.12	3.41 a ±0.29	3.66 a ±0.21	3.75 a ±0.33	3.62 a ±0.18	3.51 a ±0.23	3.7 a ±0.18
upper epidermis thickness [µm]	40.18 a ±1.1	41.07 a ±1.12	40.93 a ±1.18	40.74 ±0.86	40.94 a ±1.32	41.57 a ±0.87	40.61 a ±0.59	40.65 a ±0.89	40.38 a ±0.74	40.72 a ±1.43
lower epidermis thickness [µm]	40.18 a ±1.1	41.07 a ±1.12	40.93 a ±1.18	40.74 ±0.86	40.94 a ±1.32	41.57 a ±0.87	40.61 a ±0.59	40.65 a ±0.89	40.38 a ±0.74	40.72 a ±1.43
palisade mesophyll thickness [µm]	109.83 a ±1.17	109.95 a ±0.99	110.25 a ±1.49	108.61 a ±2.96	111.38 a ±1.58	110.07 a ±1.45	111.25 a ±1.07	111.25 a ±1.36	113.11 a ±1.63	111.59 a ±3.27
spongy mesophyll thickness [µm]	131.95 a ±2.00	132.75 a ±3.36	130 a ±2.74	132.81 a* ±2.94	128.68 a ±2.82	132.57 a* ±1.62	132.8 a ±2.17	131.75 a ±2.19	130.68 a* ±1.52	131.19 a ±3.28
area of air spaces (per cross-section) [µm^2^]	2409.7 a ±227.9	2657.8 a ±414.5	2124.4 a ±139.8	2268.7 b ±300.9	2649.1 b* ±489.0	1814.9 a** ±96.7	1438.6 b ±52.4	3141.4 a*** ±138.7	3966.3 a*** ±216.1	4021.4 a*** ±411.2

Asterisks indicate statistically significant differences between metal-treated plants and the respective control within the same ecotype: * *p* < 0.05; ** *p* < 0.01; *** *p* < 0.001. Differences between ecotypes under the same experimental conditions are indicated by letters; values followed by the same letters do not differ significantly (n = 10).

**Table 3:** Types, average number, area, and location of calcium oxalate crystals in the cross-sections of leaves of *Dianthus carthusianorum* from the metallicolous (M) and non-metallicolous (NM) ecotypes taken from the field sites or hydroponically grown under controlled conditions for 14 days in control medium (0) or in medium supplemented with 50 μM Cd, 30 μM Pb, or 500 μM Zn.

	Field Sites		Controlled Conditions							
	M	NM	M				NM			
Parameter			0	Cd	Pb	Zn	0	Cd	Pb	Zn
type of CaOx crystals	irregular shape	druses	druses	druses	druses styloids	druses	druses	druses	druses	druses
number of crystals (per cross-section) •	82.3 a ±7.0	3.2 b ±0.8	5 a 1.1	13 a*** 3.0	16 a*** 1.7	4 a 2.1	4.2 a ±1.6	9 b** ±2.4	8.2 b** ±1.5	6.8 b** ±0.8
area of crystals per cross section [µm^2^] •	646.1 a ±212.5	728.7 a ±368.8	1043.4 a ±247	2999 a*** ±1329.5	3803.3 a*** ±1834.2	3653.9 a*** ±2234.1	960.4 a ±336.7	3096.1 a*** ±1155.3	3115.2 a*** ±1308.3	2073.7 a *** ±703.7
number of crystals (per cross-section) ••	8 a ±1.8	2.7 a ±1.0	4.5 a ±1.2	6.7 a* ±1.8	6.7 a** ±0.8	8 a** ±1.7	3.5 a ±2.4	3.8 b ±2.0	2.8 b ±2.1	3.5 b ±1.4
area of crystals on cross section [µm^2^] ••	431 a ±181.9	456.9 a ± 197.7	399.7 a ± 170.2	488 a ± 254.1	353.8 b ± 158.7	487.7 a* ± 231	381.1 a ± 196.0	422.8 a ±218	491.6 a** ± 207.5	342.3 b ± 175.2

• crystals located in mesophyll tissues; •• crystals located at conductive tissues. Asterisks indicate statistically significant differences between metal-treated plants and the respective control at * *p* < 0.05; ** *p* < 0.01; *** *p* < 0.001. Two ecotypes (M and NM) were compared under the same experimental conditions using letters; values followed by the same letters do not differ significantly (n = 10).

**Table 4:** Dyes used for histochemical analyses of cross-sections.

Microscope for Analysis	Dye	Cell Structure/Compound Visualized by the Dye	Reference
light microscope	toluidine blue	cell wall, crystals	[60]
	Sudan IV	suberized cell wall, cuticle, oil droplets, crystals	[61]
	ruthenium red	pectin, collenchyma, very clearly visible crystals	[61]
	Shiff’s reagent	polysaccharides	[60]
	10% FeCl_3_ in methanol	polyphenols (tannins, lignins)	[62]
fluorescence microscope	auramine O (excitation light 400–440 mm)	cuticle, lignin, and suberin in plant cell walls	[63]
	aniline blue (excitation light 330–360 nm)	β-1,3- and 2,4-glucans, vascular bundles	[61]
	calcofluor (excitation light 365–410 nm)	cellulose, polysaccharides, β-1,3-glucans in the cell wall, pectins	[61]

## Data Availability

Data are contained within the article; further inquiries can be directed to the corresponding author.
